# Accuracy of Tools to Differentiate Single From Recurrent Fallers Pre-Frail Older Women

**DOI:** 10.3389/fpubh.2022.716851

**Published:** 2022-05-17

**Authors:** Tamires Terezinha Gallo da Silva, Jarbas Melo Filho, Simone Biesek, Audrin Said Vojciechowski, Victória Zeghbi Cochenski Borba, Anna Raquel Silveira Gomes

**Affiliations:** ^1^PhD Program in Physical Education, Federal University of Paraná, Curitiba, Brazil; ^2^Departament of Massage Under Graduation, Federal Institute of Paraná, Curitiba, Brazil; ^3^Internal Medicine Department and Masters and PhD Programs, Endocrinology Service, Hospital de Clínicas, Federal University of Paraná, Curitiba, Brazil; ^4^Masters and PhD Programs in Physical Education, Prevention and Rehabilitation in Physiotherapy Department, Federal University of Paraná, Curitiba, Brazil

**Keywords:** frail elderly (MeSH), accidental falls (MeSH), musculoskeletal system (MeSH), muscle strength (MeSH), gait (MeSH), sensitivity and specificity (MeSH)

## Abstract

**Objectives:**

The objectives of this study were to analyze and compare musculoskeletal and functional performance and present cutoff points to differentiate pre-frail community-dwelling older women regarding their fall history: non fallers (0 falls), fallers (single fall), and recurrent fallers (≥2 falls).

**Method:**

This is a cross-sectional, retrospective study on 90 pre-frail community-dwelling older women (71.2 ± 4.49 years) according to Fried criteria. We assessed peak torque (PT) (isokinetic dynamometer), muscle architecture/mass (ultrasound/dual-energy X-ray absorptometry), and the following functional performance: usual gait speed (UGS), fast gait speed (FGS), walking speed reserve (WSR), cadence and step length, and timed up and go.

**Results:**

The recurrent fallers presented lower UGS (1.12 ± 0.18 vs. 1.29 ± 0.28 m/s; *p* = 0.05) and isometric PT of knee extensors than the fallers (89.88 ± 20.99 vs. 115.55 ± 23.09 Nm; *p* = 0.01), and lower FGS than the fallers (1.35 ± 0.26 vs. 1.5 ± 0.29 m/s; *p* = 0.03) and non-fallers (1.35 ± 0.26 vs. 1.52 ± 0.26 m/s; *p* = 0.01). The outcomes that differentiated the fallers from the non-fallers were both WSR calculated as a difference (WSRdiff) (≤0.26 m/s) and WSR calculated as a ratio (WSRratio) (≤1.25 m/s), while to differentiate the recurrent fallers from the non-fallers were FGS (≤1.44 m/s) and step length (≤73 cm). The following cutoff points might be used to differentiate recurrent fallers and fallers: UGS (≤1.12 m/s), FGS (≤1.34m/s), step length (≤73 cm), PT knee extension (≤114.2 Nm), PT knee flexion (≤46.3 Nm), and PT ankle dorsiflexion (≤22.1 Nm).

**Conclusion:**

Recurrent fallers community-dwelling pre-frail older women presented a worse musculoskeletal and functional performance when compared to the non-fallers and fallers. Gait speed, step length, PT of both knee extension and flexion, and ankle dorsiflexion can be used to identify both single and recurrent fallers pre-frail older women, contributing to guide interventions and prevent falls and fractures.

## Introduction

Frailty represents a state of age-related physiological vulnerability, which results from the body's reduced ability to cope with adverse health situations, such as hospitalizations, falls, and functional reductions (1, 2). According to the frailty phenotype, a pre-frail older person presents one or two of the following criteria: unintentional weight loss, muscle weakness, exhaustion, low physical activity, and slowness and frail when three of them are found (3).

The prevalence of pre-frailty is significantly higher in older person, ranging from 49.3 to 65.3% (4, 5) when compared to frailty (16.2–19.6%) (4, 6, 7). Moreover, the prevalence of pre-frailty is higher in older women (56.3%) (4).

Frailty is associated with increased risk of falls even in non-fallers (8) and is considered a significant predictor of future falls among community-dwelling older people (9). In addition, frail and pre-frail older women present an increased risk of recurrent falls (OR = 2.41, 95% CI 1.93–3.01; OR = 1.23, 95% CI 1.02–1.48, respectively), hip fractures (RR = 1.7, 95% CI 1.35–2.15; RR = 1.34, 95% CI 1.12–1.6, respectively), and mortality (RR = 1.82, 95% CI 1.56–2.13; RR = 1.32, 95% CI 1.18–1.48, respectively) in comparison to non-frail older women (10). Furthermore, pre-frailty is associated with gait and balance impairments (9, 11), but it is still not established the musculoskeletal parameters, and functional performance cutoff points to differentiate pre-frail older women based on their fall history (non-fallers, 0 falls; fallers, single fall; recurrent fallers, ≥2 falls).

According to the World Health Organization (12), one-third of older people (>65 years) fall once a year and 5% sustain fracture. Older women fall more than older men and have higher prevalence of musculoskeletal diseases such as osteoporosis and faster muscle loss, they have more functional limitations and longer life expectancies (13–17). Maintaining skeletal muscle strong is critical to a healthy longevity throughout lifetime (17). These reasons bring the needed to investigate musculoskeletal and functional performance associated to fall history in pre-frail community-dwelling older women to guide interventions targeting this vulnerable underserved population.

Gait parameters such as usual gait speed (UGS), fast gait speed (FGS), step length, walking speed reserve (WSR) are able to discriminate older adults at the three levels of frailty but not regarding their fall history (18). On the other hand, it has been already reported that self-selected walking speed (SSWS) and maximal walking speed (MWS) discriminated fallers and non-fallers, but they did not assess levels of frailty and stratify by gender (19). Furthermore, tests such as Timed Up and Go (TUG), five times sit-to-stand test (FTSST) and handgrip strength (HS) have already been evaluated for their effectiveness in differentiating non-fallers from fallers (19–24). However, these studies stratified the samples into non-fallers (0 fall) and fallers (≥1 fall) (19, 21, 24) or into non-recurrent fallers (0 falls or≤1 fall) and recurrent fallers (≥2 falls) (20–23), failing not only to consider the difference between a single fall and recurrent falls but also to stratify by sex and physical frailty (25).

In addition, the studies have not investigated musculoskeletal function (muscle architecture and isokinetic peak torque), and the samples were healthy older person, not being possible to apply the cutoff points to pre-frail community-dwelling older women. Therefore, the aim of this cross-sectional study was to analyze and compare musculoskeletal function and functional performance, as well as determine cutoff points to differentiate pre-frail community-dwelling older women regarding their fall history, stratified into non-fallers (0 falls), fallers (single fall), and recurrent fallers (≥ 2 falls). The hypothesis of the study was that musculoskeletal function would be worse and enough to differentiate single from recurrent fallers pre-frail older women.

## Methods

### Study Design

This is a cross-sectional, retrospective study developed based on the Standards for Reporting of Diagnostic Accuracy Studies (STARD) and carried out from January 2017 to December 2018. The data bank used in this study came from the research project “Effects of physical with the Nintendo Wii Fit Plus®and protein supplementation on skeletal muscle function and the risk of falls in pre-frail older women: Protocol for a randomized controlled clinical trial (WiiProtein Study),” with the previously published evaluation protocol (26). The study was approved by the Ethics Committee for research on human of the Hospital de Clínicas da Universidade Federal do Paraná, Curitiba, Paraná, beings (certificate number 58865916.8.0000.0096; approval number 1.804.775). The participants of the study signed an informed consent form according to the World Medical Association Declaration of Helsinki.

### Participants

Older Women, pre-frail according to Fried et al. (3), that is, presenting one or two of the following criteria: (1) Weight loss; (2) Exhaustion; (3) Low level Physical Activity; (4) Decreased gait speed and (5) Low Grip Strength, ≥65 years old, living in the city of Curitiba and its metropolitan region, Paraná, Brazil. The sample was the participants included (*n* = 90) in the data bank used for the “WiiProtein study” (26). The inclusion criteria were as follows: show “moderate” kidney function [i.e., glomerular filtration rate (GFR) of 30–60 ml/min/1.73 m^2^] as estimated by the Chronic Kidney Disease Epidemiology Collaboration (CKD-EPI) equation. If a woman presents type II diabetes, it should be compensated (<8% glycated hemoglobin), and visual acuity assessed by the Snellen card considering at least (20/70 unilateral). The exclusion criteria were as follows: acute or terminal illness, metabolic instability, or decompensated cardiovascular disease, cognitive deficits as determined by the Mini-Mental State Examination considering the following cutoff points 18/19 for illiterate and 24/25 for literate (27), neurological disorders and/or traumatic-orthopedic conditions that prevent participant from carrying out the evaluations, type I diabetes, taking medication that might affect muscle metabolism (corticoids) or postural balance (anticholinergics, antihistamines, benzodiazepines, calcium channel antagonists, or dopamine receptor antagonists) (28), an hearing loss that prevents understanding verbal instructions, and any serious deficiency described in the medical records such as cardiac, respiratory, or hepatic deficiency and/or decompensated arterial hypertension (BP ≥ 140/90 mmHg), as described in the “WiiProtein study” (26).

#### Sample Size

Sample power was calculated using the F-tests Family of the G^*^Power 3.1.9 program, by one-way ANOVA, considering the sample (*n* = 90), effect size of 0.4 (mean effect), and type I error (rate of error of 5%). Thus, the sample power (1–β) of the study was 0.92.

### Fall History

**Figure 1:**
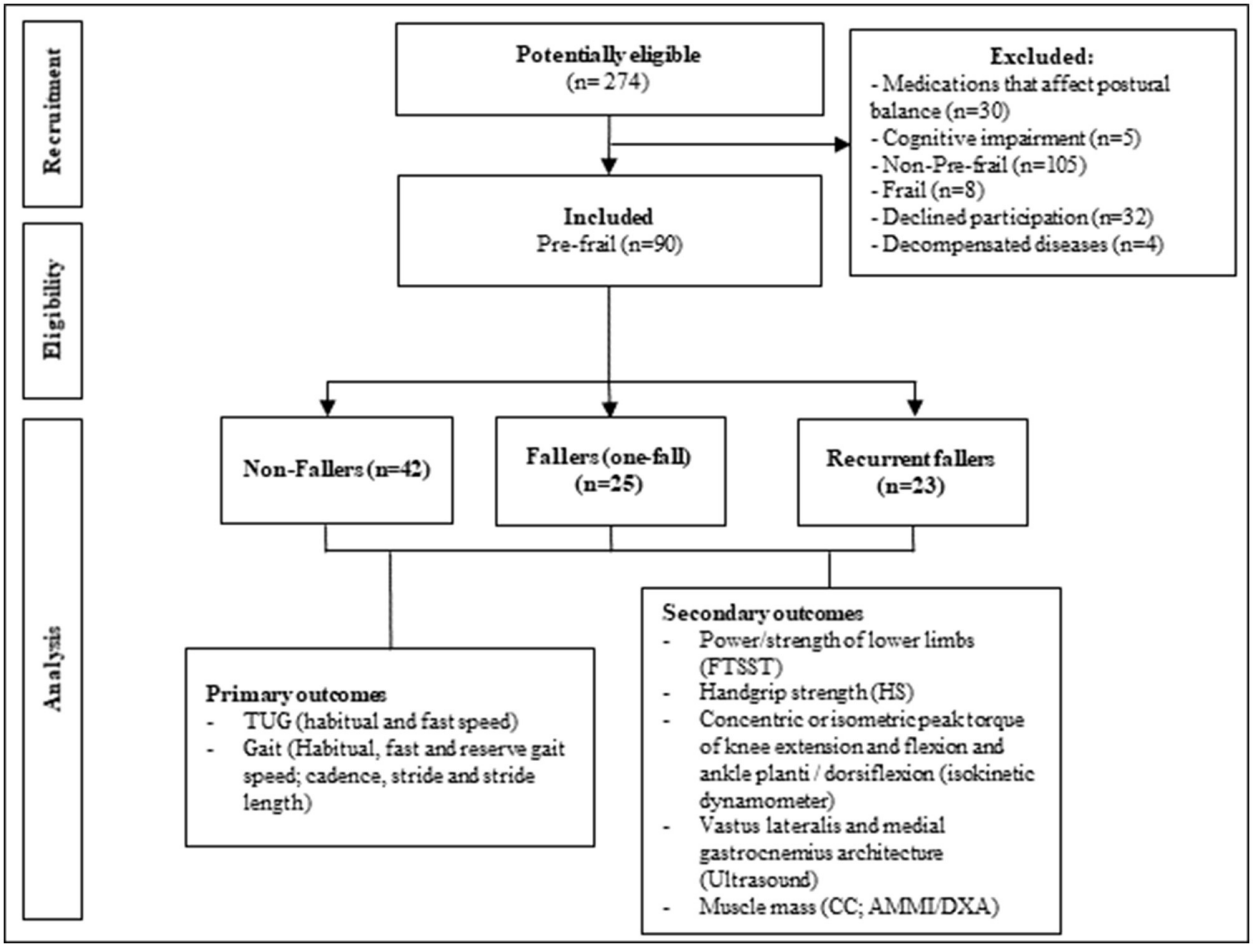
Study flow chart. FTSST, five times sit-to-stand test; HS, handgrip strength; Cc, calf circumference; AMMI, appendicular muscle mass index; DXA, dual-energy X-ray absorptiometry.

In our study, we consider a fall as any event resulting from a body change that makes an individual to inadvertently fall to the ground. This definition does not encompass the result of a violent blow, sudden paralysis, loss of consciousness, or epileptic seizure (24). The evaluation of fall history occurred through self-report, with the question “*Did you suffer any falls in the last 12 months? If so, how many times?”* The participants were also asked about the location, reasons, and consequences of the fall. According to fall history, the older women were stratified into non-fallers (NF, 0 fall), fallers (F, single fall), and recurrent fallers (RF, ≥2 falls in the last year) (25) as shown in the Figure 1. The evaluators of primary and secondary outcomes were masked about the history of falls, i.e., they did not know if a participant was a non-fallers, faller, or recurrent faller.

### Sample Characterization

To characterize the sample and the assessment protocol for general health status, the following variables were taken into consideration through self-reports: age, education, marital status, occupation, income, ethnicity, number of medications in use, number of diseases, history of fractures, hearing acuity, sphincter control, use of orthoses, use of metallic prostheses, and history of conservative and/or surgical treatments (26), body mass, and height. Body mass index (BMI) was characterized according to the following cutoff points: underweight (BMI≤23 kg/m^2^), normal weight (23 > BMI <28 kg/m^2^), pre-obesity (28≤BMI <30 kg/m^2^), and obesity (BMI ≥ 30 kg/m^2^) (26, 29).

Pain and function of the knees and hips were assessed with the Lequesne's Algofunctional Questionnaire (30), and depressive symptoms with the Geriatric Depression Scale (GDS) 15 (31).

The DXA (Lunar Prodigy Advance PA+302284, Madison, United States) and appendicular skeletal muscle mass index (ASMMI) were used to quantify bone mineral density (BMD) following the recommendations of the International Society for Clinical Densitometry (ISCD). For the diagnosis of osteoporosis, we considered the parameters established by the World Health Organization: normal bone mass (T-score ≥-1 SD), osteopenia (T-score in between −1 and −2.49 SD), and osteoporosis (T-score≤−2.5 SD) (32).

Probable sarcopenia was identified by low handgrip strength (HS <16 kg), which, if associated with low muscle mass (ASMMI <5.5 kg/m^2^), confirmed the sarcopenia. In addition, if a low functional performance was detected by gait speed (GS≤0.8 m/s), it was considered as severe sarcopenia (33).

### Primary Outcomes (Functional Tests)

Functional mobility was assessed with the TUG performed at both usual and fast gait speed according to the protocol already described in the “Wii protein study” (26).

The 10-m walk test was conducted to assess both usual (UGS) and fast gait speed (FGS) (safely executed without running). For gait assessment, four marks were drawn on the floor (1st mark: 0 m, 2nd mark: 1 m, 3rd mark: 9 m, and 4th mark: 10 m). The test started with the participant positioned at the first mark, and, after the “go” command from evaluator 1, the participant walked for a distance of 10 m in a straight line till the 4th mark where evaluator 2 was stopped. The first and last meters were discarded because they were considered participant's acceleration and deceleration phases. The test was performed three times with the older woman walking at her usual speed (UGS) and, successively, three times at fast speed (FGS). UGS was considered as the average of the three times, divided by the distance, i.e., 8 m, to calculate the UGS and FGS in m/s (34–36).

To quantify walking speed reserve (WSR), two calculations were performed: FGS subtracted from UGS (difference) (WSRdiff), and FGS divided by UGS (ratio) (WSRratio) (19).

To assess the gait parameters (step length in cm, stride length in m, and cadence in steps/min), a treadmill (Gait Trainer 2; BIODEX®) was used. The participant was instructed to walk on the treadmill for 3 min at usual speed. Two times were taken, the first for familiarization and the second for recording the values, and there was a 2-min interval between two attempts (37).

### Secondary Outcomes (Musculoskeletal Function)

Lower limb strength was assessed by the FTSST. The isometric and concentric isokinetic torques (60, 180°/s) of the knee extensors and flexors and ankle plantar/dorsiflexors were assessed with an isokinetic dynamometer (BIODEX®); System 4 Pro™; both protocols have already been described in the “Wii-protein study” (26).

The B-Mode ultrasound (Logiq Book XP; General Eletric®) with a linear-array probe (3.8 mm, 11 MHz) was used to assess muscle architecture, i.e., muscle thickness (MT), pennation angle (PA), and fascicle length (FL) of the vastus lateralis (50% of the distance between bone prominences of the greater trochanter and the lateral condyle of the femur) and the medial gastrocnemius (between 30 and 40% of the popliteal line and the medial malleolus). The probe was positioned perpendicular to the skin surface of both muscles and coated with water-soluble transmission gel, which provided acoustic contact without depressing the dermal surface. All images were analyzed using the Image J software (National Institutes of Health, Bethesda, Maryland). MT was defined as the mean distance between deep and superficial fascial planes, measured in five places along the ultrasound image; PA was defined as the angle of insertion of muscle fiber fascicles into the deep fascial plane; FL was defined as the length of the fascicular path between the insertions of the fascicle into the superior and deep fascial planes (38–43). When the end of the fascicle extended off the acquired ultrasound image, FL was estimated with a trigonometric function according to Abellaneda et al. (44).

Calf circumference (CC) was measured while the older women were seated, considering the greater prominence. The calculation of ASMMI (DXA) was performed by the addition of the lower limb and upper limbs value, divided by the height^2^ of the participant (45).

### Analysis

The primary and secondary outcomes are mentioned at Figure 1. For the comparison between the groups (non-fallers, fallers, and recurrent fallers), we conducted a one-way ANOVA test (parametric) and a Kruskal–Wallis (non-parametric) test with Bonferroni *post-hoc*; and for the categorical data a Chi-square or Fisher's exact test was conducted. To analyze the accuracy of study instruments, we calculated sensitivity (Sn), specificity (Sp), positive likelihood ratio (LR+), negative likelihood ratio (LR-), positive predictive value (PPV), and negative predictive value (NPV). Receiver operating characteristic (ROC) curves were constructed to identify the viability of the instruments (*p* < 0.05). Multivariate logistic regression was performed to verify the influence of variables (age, BMI, marital status, and number of diseases) on significant results of the ROC curves.

For the analyses, missing data were not considered. Thus, the number of the sample considered for each variable is described in the tables of the results. All the analyses were performed using Statistical Package for the Social Sciences (SPSS®) version 25, except for the analysis of the instrument's accuracy, which was performed using the MedCalc® program, with a significance level of *p*≤0.05.

## Results

### Sample Characteristics

The sample consisted of 90 pre-frail older women stratified into non-fallers [0 falls, *n* = 42 (46.7%)], fallers [single fall, *n* = 25 (27.8%)], and recurrent fallers (≥2 falls, *n* = 23 (25.6%)]. The description of the sample's clinical and sociodemographic characteristics are presented in Table 1.

**Table 1:** Comparison of the clinical, anthropometric, and sociodemographic characteristics and distributions of pre-frail non-fallers, fallers, and recurrent fallers older women.

		**Pre-Frail (Total) (*n* = 90)**	**Non-Fallers (*n* = 42)**	**Fallers (*n* = 25)**	**Recorrent fallers (*n* = 23)**	***p***
Age (years)^†^		71.20 ± 4.49; 71.0 (60–84)	70.29 ± 4.30	71.44 ± 4.75	72.61 ± 4.32	0.13
Stature (m)^∓^		1.55 ± 0.06	1.56 ± 0.07	1.56 ± 0.09	1.53 ± 0.05	0.13
Body mass (kg)^∓^		70.51 ± 12.24	70.67 ± 10.97	71.00 ± 14.12	69.70 ± 12.83	0.92
BMI (Kg/m^2^)^∓^		29.18 ± 4.27	29.01 ± 4.29	28.99 ± 4.46	29.72 ± 4.20	0.78
AA Hip Lequesne (points change for score)^†^		3.24 ± 4.49; 0.5 (0–16)	1.45 ± 2.56; 0.0 (0–8.5)	4.45 ± 4.35; 3.2 (0–14)	2.55 ± 2.96; 6.5 (0–8.5)	0.82
AA knee Lequesne (points change for score)^†^		3.37 ± 4.53; 1.7 (0–17)	1.54 ± 2.17; 0.0 (0–6.5)	3.65 ± 4.44; 2.7 (0–14)	6.58 ± 6.67; 6.0 (0–17)	0.14
Number of medications (*n*)^†^		4.14 ± 2.66; 4.0 (0–12)	4.00 ± 2.49; 3.50 (0–10)	4.88 ± 2.47; 5.0 (1–9)	3.61 ± 3.07; 3.0 (0–12)	0.13
Number of diseases (*n*)^†^		2.44 ± 1.30; 2.0 (0–5)	2.45 ± 1.19; 2.0 (0–5)	2.92 ± 1.38; 3.0 (0–5)^a^	1.91 ± 1.27; 2.0 (0–4)	0.03^*^
MMSE (score)^†^		27.3 ± 2.44; 28.0 (21–30)	27.67 ± 2.11; 28.0 (22–30)	27.12 ± 2.89; 28.0 (21–30)	27.74 ± 2.54; 28.0 (21–30)	0.74
Geriatric depression scale-GDS^†^		3.94 ± 0.29; 3.0 (0–12)	3.59 ± 2.81; 3.0 (0–11)	4.12 ± 2.72; 3.0 (1–12)	4.39 ± 2.69; 4.0 (1–11)	0.26
Ethnicity^‡^	Black	7.8% (7)	2.4% (1)	8.0% (2)	17.4% (4)	0.08
	White	77.8% (70)	83.3% (35)	68.0% (17)	78.3% (18)	
	Non-White	14.4% (13)	14.3% (6)	24.0% (6)	4.3% (1)	
Visual acuity^‡^	Normal vision	13.3% (12)	11.9% (5)	24.0% (6)	4.3% (1)	0.09
	Using correction	86.7% (78)	88.1% (37)	76.0% (19)	95.7% (22)	
Hearing acuity^‡^	Normal hearing	60.0% (54)	69.0% (29)	52.0% (13)	52.2% (12)	0.26
	Hearing loss	40.0% (36)	31.0% (13)	48.0% (12)	47.8% (11)	
Educational level^‡^	Illiterate	2.2% (2)	–	4.0% (1)	4.3% (1)	0.72
	1–4 years	38.9% (35)	45.2% (19)	32.0% (8)	34.8% (8)	
	5–8 years	21.1% (19)	21.4% (9)	24.0% (6)	17.4% (4)	
	>8 years	37.8% (34)	33.3% (14)	40.0% (10)	43.5% (10)	
Marital status^‡^	Married	45.6% (41)	50.0% (21)	52% (13)	30.4% (7)	0.04^*^
	Divorced	6.7% (6)	9.5% (4)	4.0% (1)	4.3%(1)	
	Separated	6.7% (6)	2.4% (1)	4.0% (1)	17.4% (4)	
	Widow	33.3% (30)	38.1% (16)	28.0% (7)	30.4% (7)	
	Single	7.8% (7)	–	12.0% (3)	17.4% (4)	
Occupation^‡^	Retired with another occupation	21.1% (19)	21.4% (9)	16.0% (4)	21.7% (5)	0.51
	Retired without other occupation	47.8% (43)	38.1% (16)	64.0% (16)	52.2%(12)	
	Domestic work	26.7% (24)	35.7% (15)	16.0% (4)	21.7% (5)	
	Outdoor jobs	4.4% (4)	4.8% (2)	4.0% (1)	4.3% (1)	
Income^‡^	Retirement	73.3% (66)	64.3% (27)	88.0% (22)	73.9% (17)	0.56
	Pension	14.4% (13)	16.7% (7)	4.0% (1)	21.7% (5)	
	Children's allowance beneficiaries	1.1% (1)	2.4% (1)	–	–	
	Rental	1.1% (1)	2.4% (1)	–	–	
	Work	2.2% (2)	2.4% (1)	4.0% (1)	–	
	Retirement and pension	3.3% (3)	7.1% (3)	–	–	
	Others	4.4% (4)	4.8% (2)	4.0% (1)	4.3% (1)	
Dwelling^‡^	Alone	22.2% (20)	26.2% (11)	12.0% (3)	21.7% (5)	0.31
	Husband	35.6% (32)	38.1% (16)	40.0% (10)	26.1% (6)	
	Children	24.4% (22)	21.4% (9)	28.0% (7)	30.4% (7)	
	Other family members	11.1% (10)	4.8% (2)	12.0% (3)	21.7% (5)	
	Husband and children	6.7% (6)	9.5% (4)	4.0% (1)	–	
Urinary incontinence^‡^	Yes	38.9% (35)	38.1% (16)	40.0% (10)	39.1% (9)	0.98
	No	61.1% (55)	61.9% (26)	60.0% (15)	60.9% (14)	

*BMI, body mass index; AA, algofunctional assessment; MMSE, Mini-Mental State Examination*;

† *Mean values ± standard deviation; median (minimum–maximum) compared by Kruskal–Wallis test*.

∓ *Mean values ± standard deviation compared by one-way ANOVA Test*.

‡ *Relative (%) and absolute (number) frequency values by chi-square or Fisher's exact test*.

* *Significant difference*;

a *Significantly different from the recurrent fallers group, p = 0.03*.

**Table 2:** Characterization and comparison of the functional performance of pre-frail non-fallers, fallers, and recurrent fallers older women.

	***n***	**Pre-Frail (*n* = 90)**	***n***	**NF (0 falls)**	***n***	**F (single fall)**	***n***	**RF (≥2 falls)**	***p***
TUGU (s)	89	9.90 ± 3.32–9.28 (6.8–33.6)	42	9.47 ± 2.00–9.00 (6.82–18.26)	25	10.58 ± 5.12–9.32 (6.87–33.66)	22	10.04 ± 2.72–9.76 (6.95–19.15)	0.54
TUGF (s)	87	8.13 ± 2.17–7.78 (5.56–23.1)	41	7.88 ± 1.39–7.48 (5.94–11.29)	25	6.95 ± 3.45–7.77 (5.56–23.19)	21	8.34 ± 1.34–8.38 (5.93–10.72)	0.29
UGS (m/s)	88	1.22 ± 0.23	41	1.23 ± 0.21	25	1.29 ± 0.28^a^	22	1.12 ± 0.18	0.05^*^
FGS (m/s)	88	1.47 ± 0.27–1.45 (0.63–2.38)	41	1.52 ± 0.26–1.54 (1.09–2.38)^b^	25	1.50 ± 0.29–1.45 (0.63–2.06)^c^	22	1.35 ± 0.26–1.30 (0.88–1.95)	0.04^*^
WSRdiff (m/s)	88	0.25 ± 0.13–0.23 (0.0–0.65)	41	0.29 ± 0.13–0.27 (0.04–0.65)	25	0.21 ± 0.11–0.21 (0.00–0.45)	22	0.22 ± 0.13–0.22 (0.03–0.56)	0.07
WSRratio (m/s)	88	1.21 ± 0.11–1.19 (0.99–1.64)	41	1.24 ± 0.12–1.22 (1.03–1.64)	25	1.17 ± 0.09–1.17 (0.99–1.50)	22	1.20 ± 0.11–1.19 (1.03–1.48)	0.058
LEN stride (m)	54	1.10 ± 0.23	25	1.10 ± 0.24	18	1.15 ± 0.23	11	1.01 ± 0.24	0.36
LEN step (cm)	54	68.59 ± 14.83	25	69.72 ± 16.07	18	72.00 ± 14.68	11	60.45 ± 9.08	0.10
CAD (steps/min)	54	106.65 ± 14.80–105.0 (46.00–134.67)	25	105.8 ± 17.49–104.7 (46–134.67)	18	109.37 ± 11.91–111.6(84.33–131)	11	104.15 ± 12.76–102.67 (82.67–124.33)	0.49

*Values expressed as mean ± standard deviation - median (minimum–maximum)*.

* *Significant difference*;

a *Significantly different from the recurrent fallers group, p = 0.05)*;

b *Significantly different from the recurrent fallers group, p = 0.01)*;

c *Significantly different from the recurrent fallers group, p = 0.03; TUGU, Timed-Up-and-Go at usual speed; TUGF, the Timed-Up-and-Go at fast speed; UGS, usual gait speed; FGS, fast gait speed; WSRdiff, walking speed reserve calculated by the difference (FGS-UGS); WSRratio, walking speed reserve calculated by the ratio (FGS/UGS); n, number of older women; LEN, length; CAD, cadence; NF, non-fallers; F, fallers; RF, recurrent fallers*.

Regarding the frequency of physical frailty, low HS was the most present criterion in the total sample (58.9%), among non-fallers older women (64.3%), and among recurrent fallers older women (65.2%). The most frequent criterion in the group of fallers (single fall) was exhaustion/fatigue (48.0%) (Supplementary Table 1).

The densitometric characteristics and frequencies of sarcopenia in the sample, with no difference between groups, are shown in Supplementary Table 2.

More than half [52% (*n* = 13)] of the single falls occurred indoors. Recurrent falls varied between locations, with 17.4% (*n*= 4) reported falling only indoors and 17.4% (*n* = 4) reported falling both indoors and outdoors such as in the backyard or outside the house in known locations. The most common cause of single falls and recurrent falls were trips [36% (*n* = 9) and 30.4% (*n* = 7), respectively], and their consequences were contusions, 40% (*n* = 10) in the single fallers and 60.9% (*n* = 14) in the recurrent fallers.

### Comparison Between Groups

A reduction in UGS was observed in the recurrent fallers when compared to the fallers (1.12 ± 0.18 vs. 1.29 ± 0.28 m/s; *p* = 0.05). Similarly, a decrease in FGS was observed in the recurrent fallers when compared to the non-fallers (1.35 ± 0.26 vs. 1.52 ± 0.26 m/s; *p* = 0.01) and the fallers (1.35 ± 0.26 vs. 1.5 ± 0.29 m/s; *p* = 0.03). In addition, a decline in the isometric PT of the knee extensors was detected when the recurrent fallers were compared to the fallers (89.88 ± 20.99 vs. 115.55 ± 23.09 Nm; *p* = 0.01; Tables 1–3).

**Table 3:** Characterization and comparison of muscle force/torque in non-fallers, fallers, and recurrent fallers pre-frail older women.

	***n***	**Pre-Frail (*n* = 90)**	***n***	**NF (0 falls)**	***n***	**F (single falls)**	***n***	**RF (≥2 falls)**	***p***
FTSST (s)	89	11.84 ± 4.00–10.92 (5.8–33.9)	42	11.92 ± 4.49–10.88 (6.10–33.91)	25	11.33 ± 2.52–11.70 (7.06–16.50)	22	12.27 ± 4.46–10.71 (5.80–27.26)	0.96
HS (Kgf)	90	19.81 ± 5.66–18.83 (1.00–34.33)	42	19.70 ± 5.66–18 (9.66–34.00)	25	20.70 ± 6.25–18.66 (10.66–34.33)	23	19.02 ± 5.05–19.66 (1.00–26.66)	0.75
**ISO peak torque (Nm)**									
Knee FLE	64	41.24 ± 11.87	30	40.03 ± 12.89	19	45.94 ± 12.11	16	38.73 ± 8.22	0.13
Knee EXT	65	102.04 ± 27.90–99.60 (28.6–149.7)	30	99.97 ± 30.88–96.40 (36.10–149.70)	19	115.55 ± 23.09–124.10 (63.50–144.20)^a^	16	89.88 ± 20.99–96.10 (28.60–114.20)	0.02^*^
DORSI ankle	66	23.47 ± 6.03	30	23.29 ± 5.88	19	25.67 ± 4.76	17	21.34 ± 7.00	0.09
PLANT ankle	66	65.67 ± 20.46	30	62.04 ± 22.47	19	73.42 ± 19.91	17	63.43 ± 15.43	0.14
**CONC peak torque (Nm)−60** **°** **/s**									
Knee FLE	89	36.47 ± 12.41–38.20 (4.4–62.2)	42	36.45 ± 13.26–40.15 (11.10–62.20)	25	38.02 ± 12.61–39.60 (4.40–55.90)	22	34.73 ± 10.77–34.15 (17.20–55.50)	0.40
Knee EXT	89	82.22 ± 23.55	42	82.93 ± 25.52	25	86.95 ± 25.51	22	75.50 ± 20.61	0.24
DORSI ankle	89	18.10 ± 3.82–17.50 (11.4–29.7)	42	18.28 ± 3.7–17.85 (11.60–29.70)	25	18.44 ± 3.91–18.00 (11.40–25.30)	22	17.37 ± 3.85–17.30 (11.50–29.50)	0.56
PLANT ankle	89	41.02 ± 14.35	42	42.52 ± 15.90	25	41.33 ± 13.33	22	37.82 ± 12.25	0.46
**CONC peak torque (Nm)−180** **°** **/s**									
Knee FLE	89	31.53 ± 10.05	42	32.01 ± 10.00	25	32.81 ± 9.77	22	29.15 ± 10.49	0.42
Knee EXT	89	57.58 ± 15.17	42	58.58 ± 15.28	25	59.28 ± 15.63	22	53.74 ± 14.45	0.39
DORSI ankle	88	16.01 ± 4.21–14.65 (10.2–29.5)	42	16.60 ± 4.38–15.70 (11.40–29.50)	24	15.44 ± 3.46–14.45 (10.20–25.80)	22	15.51 ± 4.61–14.25 (10.70–27.80)	0.32
PLANT ankle	88	24.05 ± 8.13	42	24.64 ± 9.11	24	24.12 ± 7.23	22	22.84 ± 7.23	0.70

*Values expressed as mean ± standard deviation-median (minimum–maximum)*.

* *Significant difference*;

*^a^Significantly different from the recurrent fallers group, p = 0.01; FTSST, five times sit-to-stand; HS, handgrip strength; PT, peak torque; ISO, isometric; FLE, flexion; EXT, extension; CONC, concentric; DORSI, dorsiflexion; PLANT, plantarflexion; n, number of older women; NF, non-fallers; F, fallers; RF, recurrent fallers*.

The variables of muscle architecture and mass did not present statistically significant differences between the groups (Table 4).

**Table 4:** Characterization and comparison of muscle architecture and mass in pre-frail non-fallers, fallers, and recurrent fallers older women.

	***n***	**Pre-Frail (*n* = 90)**	***n***	**NF (0 falls)**	***n***	**F (single fall)**	***n***	**RF (≥2 falls)**	***p***
FL VL (cm)	88	6.74 ± 1.09	42	6.82 ± 1.12	24	6.68 ± 1.18	22	6.64 ± 0.95	0.78
PA VL (°)	88	14.18 ± 1.91–14.06 (9.37–19.17)	42	14.26 ± 1.76–14.00 (10.90–18.19)	24	13.81 ± 1.62–13.89 (9.37–15.60)	22	14.45 ± 2.45–14.43 (9.61–19.17)	0.60
MT VL (cm)	90	1.66 ± 0.31	42	1.70 ± 0.29	25	1.62 ± 0.33	23	1.64 ± 0.32	0.56
FL MG (cm)	68	3.00 ± 0.52–2.90 (1.85–4.90)	34	3.08 ± 0.53–2.97 (2.33–4.90)	18	3.00 ± 0.57–2.95 (1.85–4.50)	16	2.84 ± 0.45–2.90 (2.16–3.48)	0.49
PA MG (°)	68	26.62 ± 3.91	34	25.76 ± 3.74	18	27.72 ± 3.70	16	27.19 ± 4.29	0.18
MT MG (cm)	88	1.35 ± 0.19–1.31 (1.04–1.82)	42	1.34 ± 0.17–1.29 (1.04–1.73)	24	1.42 ± 0.24–1.43 (1.09–1.82)	22	1.29 ± 0.17–1.27 (1.08–1.75)	0.16
CC (cm)	89	36.54 ± 3.75–35.50 (29.8–49.0)	42	36.04 ± 3.71–35.65 (29.80–44.60)	25	36.93 ± 3.64–36.50 (30.00–43.50)	22	37.04 ± 3.98–36.50 (31.00–49.00)	0.53
ASMMI (Kg/m^2^)	87	6.45 ± 0.83–6.34 (4.92–9.09)	40	6.42 ± 0.84–6.21 (5.03–9.09)	24	6.60 ± 0.82–6.63 (4.92–7.91)	23	6.35 ± 0.83–6.07 (5.20–8.75)	0.34

*Values expressed as mean ± standard deviation-median (minimum–maximum). FL, fascicle length; PA, pennation angle; MT, muscle thickness; VL, vastus lateralis; MG, medial gastrocnemius; CC, calf circumference; ASMMI, appendicular skeletal muscle mass index; n, number of older women; NF, non-fallers; F, fallers; RF, recurrent fallers*.

### Accuracy of Tests to Differentiate Single Fallers From Recurrent Fallers

The ROC analysis were run with all the variables, but we reported only variables that were significant. The ROC curves are shown in Supplementary Figure 1. The multivariate logistic regression did not show a significant difference when the covariates were considered (Table 5).

**Table 5:** Predictive values for fall risk and recurrent falls, areas under the curve (receiver operating characteristic, ROC) and risk statistics.

**Test**	**Cut-Off point**	**Sn (%)**	**Sp (%)**	**PPV (%)**	**NPV (%)**	**LR+**	**LR-**	**AUC (95% CI)**	***p* AUC value**	**MLR**
WSRdiff (m/s)^†^	≤0.26	76.0	53.7	50.0	78.6	1.64	0.45	0.643^*^ (0.506–0.779)	0.04	0.99
WSRratio (m/s)^†^	≤1.25	92.0	41.5	48.9	89.5	1.57	0.19	0.673^*^ (0.540–0.806)	0.01	0.99
FGS (m/s)^♠^	≤1.44	72.7	65.9	53.3	81.8	2.13	0.41	0.677^*^ (0.532–0.823)	0.01	0.98
LEN step (cm)^♠^	≤73	100.0	48.0	45.8	100.0	1.92	0.00	0.704^*^ (0.535–0.872)	0.01	0.98
UGS (m/s)^∓^	≤1.12	59.1	80.0	72.2	69.0	2.95	0.51	0.722^*^ (0.572–0.872)	0.00	0.96
FGS (m/s)^∓^	≤1.34	59.1	80.0	72.2	69.0	2.95	0.51	0.687^*^ (0.531–0.844)	0.01	0.98
LEN step (cm)^∓^	≤73	100.0	50.0	55.0	100.0	2.00	0.00	0.722^*^ (0.536–0.908)	0.01	0.96
ISO PT knee EXT (Nm)^∓^	≤114.2	100.0	57.9	66.7	100.0	2.37	0.00	0.806^*^ (0.656–0.956)	0.00	0.86
ISO PT knee FLE (Nm)^∓^	≤46.3	93.7	57.9	65.2	91.7	2.23	0.11	0.707^*^ (0.523–0.891)	0.02	0.99
ISO PT DORSI ankle (Nm)^∓^	≤22.1	58.8	84.2	76.9	69.6	3.73	0.49	0.707^*^ (0.531–0.884)	0.02	0.99

† *Capacity to differentiate fallers (single fall) from non-fallers older women*;

♠ *Capacity to differentiate recurrent fallers from non-fallers*;

∓ *Capacity to differentiate recurrent fallers from fallers (single fall); Sn, sensitivity; Sp, specificity; PPV, positive predictive value; NPV, negative predictive value; LR+, likelihood ratio +; LR-, likelihood ratio -; AUC, area under the curve; CI, confidence interval; MLR, multivariate logistic regression; ROC, receiver operating characteristic; WSRdiff, walking speed reserve calculated by the difference (FGS-UGS); WSRratio, walking speed reserve calculated by the ratio (FGS/UGS); FGS, fast gait speed; LEN, length; UGS, usual gait speed; PT, peak torque; ISO, isometric; FLE, flexion; EXT, extension; DORSI, dorsiflexion*;

* *(p <0.05)*.

#### Capacity to Differentiate Fallers (Single Fall) From Non-Fallers Older Women

WSRdiff and WSRratio differentiated the fallers from the non-fallers. The Sn and Sp in the ROC curve indicated the cutoff point of≤0.26 m/s for WSRdiff (Sn = 76%, Sp = 53.7%), and≤1.25 m/s for WSRratio (Sn = 96%, Sp = 41.5%). The older women who had a single 239 fall in the last year presented 1.64 times (LR+=1.64) more chance to show a positive test for WSRdiff 240 and 1.57 times for WSRratio in comparison to the non-fallers (Table 5).

#### Capacity to Differentiate Recurrent Fallers From Non-Fallers

FGS and step length differentiated the recurrent fallers from the non-fallers. The analysis of the Sn and Sp values in the ROC curve indicated the cutoff point of≤1.44 m/s for FGS (Sn = 72.7%, Sp = 65.9%) and≤73 cm for step length (Sn = 100%, Sp = 48%). The recurrent fallers older women had 2.13 times (LR+ = 2.13) of having a positive test for WSR and 1.92 times (LR + =1.92) for step length (Table 5) in comparison to the non-fallers.

#### Capacity to Differentiate Recurrent Fallers From Fallers (Single Fall)

UGS, FGS, step length, and isometric PT of knee extension, isometric PT of knee flexion, and isometric PT of ankle dorsiflexion differentiated the recurrent fallers from the fallers. The Sn and Sp in the ROC curve indicated the cutoff point of≤1.12 m/s for UGS (Sn = 59.1%, Sp = 80%),≤1.34 m/s for FGS (Sn = 59.1%, Sp = 80%),≤73 cm for step length (Sn = 100%, Sp = 50.0%),≤114.2 Nm for knee extension isometric PT (Sn = 100%, Sp =5 7.9%),≤46.3 Nm for knee flexion isometric PT (Sn = 93.7%, Sp = 57.9%), and≤22.1 Nm for ankle dorsiflexion isometric PT (Sn = 58.8%, Sp = 84.2%) (Table 5).

The older women who fell 2 or more times in the last year, when compared to the single fallers, had 2.95 times (LR+= 2.95) the chance of having a positive test for UGS and FGS, 2 times (LR + = 2) for step length, 2.37 times (LR+ = 2.37) for knee extension isometric PT, 2.23 times (LR+ = 2.23) for knee flexion isometric PT, and 3.73 times (LR+ = 3.73) for ankle dorsiflexion isometric PT.

## Discussion

The musculoskeletal function and physical performance of the pre-frail recurrent fallers older women were worse than those of the non-fallers and single fallers. Both the gait and the torque of the lower limbs were sufficient to differentiate the pre-frail fallers from both the non-fallers and the recurrent fallers, as well as to differentiate the non-fallers from the recurrent fallers.

Pre-frail older people had smaller step lengths, larger base of support, lower single support percentage, and higher double support percentage than the non-frail ones. History of falls was correlated to lower gait speed and smaller step length in pre-frail older adults (46). In addition, pre-frail older people with slow gait speed are 10.50 times more likely to become frail (47). Our study is in line with the reported data but has filled an important gap, showing that the recurrent pre-frail fallers presented a UGS slower than that of the fallers and an FGS slower than that of the fallers and non-fallers.

Moreover, our outcomes have a significant clinical application, as pre-frailty can progress into a state of frailty or revert to a non-frail state, and recurrent fallers have more chance to fall again and present an increased risk of fractures (48). Therefore, as unaddressed risk factors for falls lead to recurrent falls and poor quality of life and recurrent falls are usually due to multiple factors, the identification of gait and musculoskeletal parameters by multidisciplinary assessments might help to tailor a multicomponent intervention to target pertinent risk factors and prevent future falls and fractures in community-dwelling pre-frail older women (49).

Most older people do not report falls to physicians unless they are injured; they are recognized as “silent fallers.” Thus, this study brings an important contribution for healthcare professionals, giving cutoffs based on an easy and validated clinical tool, gait speed, to monitor and differentiate pre-frail recurrent fallers older women from non-fallers and single fallers (47, 49).

Our results also showed that the recurrent fallers had lower knee extension isometric peak torque than the single fallers. Regarding the evaluation of community-dwelling older people, a similarity was observed in the muscle strength of knee extension and flexion (60 and 180°/s) of both the non-fallers and the fallers (≥1 fall) (50). Conversely, another study observed that fallers of both sexes had lower values of peak torque (60 and 180°/s) of both knee extensors and flexors than non-fallers (51). However, these studies (50, 51) were not conducted on pre-frail older women. Majority of falls occur during walking (52), and the greater muscle strength of knee extensors is directly involved in gait parameters, such as increased speed, cadence, and step length (53). Our findings reinforce the need of knee extensor strength training not only for single fallers but also for recurrent fallers.

### Accuracy of Tests Under Study

WSRdiff and WSRratio accurately differentiated the fallers (single fall) from the non-fallers. The ability to increase the speed of walking on demand is indispensable for safe walking. Individuals who present difficulties under this demand are unable to respond to environmental stimuli such as crossing the street when the traffic lights start to flash (54). These findings strengthen the use of gait speed to identify the risk of falling. Nevertheless, WSRdiff and WSRratio were not yet reported in pre-frail older women. Thus, our data suggest that primary healthcare professionals not only assess gait speed but also calculate WSRdiff (≤0.26 m/s) and WSRratio (≤1.25 m/s) using the cutoff points obtained in this study to differentiate fallers from non-fallers community-dwelling pre-frail older women.

In our study, FGS and step length enabled the differentiation between both recurrent fallers from non-fallers and recurrent fallers from single fallers, corroborating a study that observed shorter step lengths in pre-frail older people compared to those who were non-frail, and a negative correlation with history of falls (46). Furthermore, our results reinforce a study that reported that SSWS and MWS should benefit fall risk assessments for older people (19). Thus, our data allow for us to recommend not only UGS but also FGS to assess fall risks, as they are easy to apply clinically, as well as step length, and they can be considered as predictors of recurrent falls in pre-frail older women.

UGS showed a good capacity (AUC = 0.722, 95% CI 0.572–0.872) in differentiating recurrent fallers from single fallers. In a study on community-dwelling older people, a cutoff point of 0.99 m/s was found for the risk of falls (≥1 fall), with Sn of 56% and Sp of 56% (24). Likewise, another study on community-dwelling older people found an AUC of 0.69 (95% CI 0.62–0.76), with a cutoff point of 0.76 m/s for the risk of falls (≥1 fall), showing a Sn of 65.4% and SP of 70.9% (19). The comparison of our study to others in the literature is limited, because they did not stratify their samples by sex and did not assess physical frailty. Moreover, our study suggests a cutoff point for the risk of recurrent falls by the assessment of UGS for older women who had already experienced a single fall in the last year, which is different from other studies that evaluated the risk of falls (≥1 fall) only in non-fallers older women (19, 24).

Therefore, our study confirmed the initial hypothesis that functional performance, assessed by gait speed, was enough to differentiate single from recurrent fallers pre-frail older women. Additionally, our outcomes are different from the previous studies because they investigated older people both genders with different clinical characteristics and history of falls, preventing to extrapolate the data for pre-frail older women (10, 19). The investigation of recurrent fallers are very relevant clinically, because they get fractured and hospitalized more frequently than single fallers or non-pre-frail people, impairing their functionality and independence and increasing costs and mortality (48, 55).

The isometric peak torques of both knee extension and flexion (AUC = 0.806, 95% CI 0.656–0.956); AUC = 0.707, 95% CI 0.523–0.891, respectively), as well as ankle dorsiflexion (AUC = 0.707, 95% CI 0.531–0.884), presented the best diagnostic accuracy (AUC) among the tests investigated in our study regarding the differentiation of recurrent fallers from single fallers older women. The study by Garcia et al. (56), with a sample composed of community-dwelling older women with low BMD, in which 44.8% of them were pre-frail, demonstrated that reduction in the PT of hamstrings (OR = 0.975, 95% CI 0.952–0.999) is associated with new falls as well as recurrent falls (OR = 0.983, 95% CI 0.967–0.999). Community-dwelling non-fallers older women have a knee flexion peak torque (21%) and an extension (14%) greater than those of fallers older women (57). In addition, other authors reported that the motor activation time of the anterior tibial muscle during ankle dorsiflexion explains 19.4% of the falls; and with the inclusion of the extension torque data, the model explained 27.8% of the falls. This means that the community-dwelling fallers older women have less capacity to produce torque and muscle recruitment in knee flexion and extension and ankle dorsiflexion (57). Therefore, assessing and training the muscle strength of the lower limbs is one of the main therapies to prevent falls (58).

### Strengths and Limitations

We consider a strong point of our study the indication of the importance of multidimensional assessments, seeking to identify the risk factors involved in falls and their recurrence, in order to guide interventions for pre-frail community-dwelling older women. In addition, the results of our study might be used by primary healthcare professionals as a guide for the applicability of simple and low-cost clinical tests, using the cutoff points that we found, in order to avoid negative outcomes such as fractures, hospitalizations, and other comorbidities triggered by falls mainly in pre-frail older women.

The limitations of our study consist of its cross-sectional, retrospective design that does not allow to establish a cause-effect relationship; it might be suggested to conduct a prospective longitudinal study. Also, the self-reported history of falls might have a memory bias. However, this bias may have been minimized by the intentional restriction of including in the sample only community-dwelling older women without cognitive impairment. Also, there was no control group of non-frail older women. However, the main focus of our study consisted of the subgroups (non-fallers, fallers, and recurrent fallers). Therefore, our control group consisted of non-fallers older women.

### Implications for Clinical Practice

FGS (≤1.44 m/s) and step length (≤73 cm) were able to differentiate recurrent fallers from both single fallers and non-fallers. Our results indicated that the assessment of gait speed enabled the differentiation between recurrent fallers and both single fallers and non-fallers, once the older women who are recurrent fallers presented a greater decline in FGS. In addition, the assessment of gait speed is simple and easy to apply even in basic health services.

Also, walking speed reserve (WSRdiff) (≤0.26 m/s) and walking speed reserve ratio (WSRratio) (≤1.25 m/s) should be considered for clinical practice even in places like primary health and domiciliary, because they do not require much space or technology to carry out and calculate.

Recurrent fallers presented lower gait speed and weaker knee extensor strength than fallers, bringing useful clinical information to target these musculoskeletal and functional factors involved in falls to develop physical programs, mainly for community-dwelling pre-frail older women.

## Conclusion

Recurrent fallers community-dwelling pre-frail older women presented worse musculoskeletal (lower peak torque of knee extensors) and functional performance (lower UGS and FGS) than fallers. Simple tests such as WSR (FGS subtracted from UGS, WSRdiff); and its ratio (WSRratio); FGS and step length should be recommended for assessing fall status in community-dwelling pre-frail older women. WSRdiff≤0.26 and WSRratio≤1.25 m/s might be used to differentiate pre-frail older women fallers from non-fallers. FGS≤1.44 m/s and step length≤73 cm differentiated recurrent fallers from non-fallers, and FGS≤1.34 m/s and step length≤73 cm differentiated recurrent fallers from fallers. Pre-frail older women identified with scores lower than the demonstrated cutoff points may benefit from additional fall risk assessments.

## Data Availability Statement

The original contributions presented in the study are included in the article/Supplementary Material, further inquiries can be directed to the corresponding author.

## Ethics Statement

The studies involving human participants were reviewed and approved by Ethics Committee for research in human beings at the clinical hospital of the Federal University of Paraná. The patients/participants provided their written informed consent to participate in this study.

## Author Contributions

TG: conceptualization, formal analysis, investigation, writing (review and editing), and methodology. JM: software and investigation. SB and AV: conceptualization and investigation. VB: resources and methodology. AG: conceptualization, writing (review and editing) supervision, and project administration. All authors revised the article critically and approved the final version.

## Funding

This study was financed partially by the Coordination for the Improvement of Higher Education Personnel-Brazil (CAPES)–(financial code 001), Conselho Nacional de Desenvolvimento Científico e Tecnológico (process number 306179/2016-4), and Pró-Reitoria de Pesquisa e Pós-graduação (PRPPG) at the Federal University of Paraná (UFPR) for the payment of the publication fee.

## Conflict of Interest

The authors declare that the research was conducted in the absence of any commercial or financial relationships that could be construed as a potential conflict of interest.

## Publisher's Note

All claims expressed in this article are solely those of the authors and do not necessarily represent those of their affiliated organizations, or those of the publisher, the editors and the reviewers. Any product that may be evaluated in this article, or claim that may be made by its manufacturer, is not guaranteed or endorsed by the publisher.

## Abbreviations

GS: gait speed
UGS: usual gait speed
FGS: fast gait speed
PT: peak torque
WSR: walking speed reserve
Nm: Newton meter
FTSST: five times sit-to-stand test
TUG: Timed Up and Go
MMSE: Mini Mental State Examination
DXA: dual-energy X-ray absorptometry
MT: muscle thickness
PA: pennation angle
FL: fascicle length
VL: vastus lateralis
MG: medial gastrocnemius
diff: difference
Sn: sensitivity
Sp: Specificity
NF: non-fallers
F: fallers
RF: recurrent fallers
cm: centimeter.
