# Supplementary material for: Accuracy of Tools to Differentiate Single From Recurrent Fallers Pre-Frail Older Women
Source: Front Public Health. 2022 May 17;10:716851. doi: 10.3389/fpubh.2022.716851 (PMC9152280; doi:10.3389/fpubh.2022.716851)
Supplement: Supplementary file 1 [file Data_Sheet_1.pdf]

Supplementary Material

Contents

Appendix 1: Suplementary Table 1. comparison of the frailty criteria frequency on the non-fallers, fallers and recurrent fallers older women.

Appendix 2: Suplementary Table 2. classification and comparison of bone mineral density and frequency of sarcopenia in pre-frail non-fallers, fallers and recurrent fallers.

Appendix 3: Suplementary Figure 1. ROC curves.

APPENDIX 1: TABLE 1. COMPARISON OF THE FRAILTY CRITERIA FREQUENCY ON THE NON-FALLERS, FALLERS AND RECURRENT FALLERS OLDER WOMEN.

		Pre-frail (Total) (n=90)	Non-fallers (0 falls) (n=42)	Fallers (n = 25)	Recurrent fallers (≥2falls) (n=23)	p
**Weight loss**		17.8 (16)	16.7 (7)	28.0 (7)	8.7 (2)	0.22
**Exhaustion/Fatigue**		45.6 (41)	45.2 (19)	48.0 (12)	43.5 (10)	0.95
**Low HS**		58.9 (53)	64.3 (27)	44.0 (11)	65.2(15)	0.20
**Low GS4**		3.3 (3)	-	4.0 (1)	8.7 (2)	0.88
**Low calorie expenditure**		3.3 (3)	2.4 (1)	4.0 (1)	4.3 (1)	0.89
**Total of frailty criteria**	1	71.1 (64)	69.0 (29)	72.0 (18)	73.9 (17)	0.91
	2	28.9 (26)	31.0 (13)	28.0 (7)	26.1 (6)	

Relative in percentage and absolute (number) frequency values compared by Chi-square or Fisher's exact test.

APPENDIX 2: TABLE 2. CLASSIFICATION AND COMPARISON OF BONE MINERAL DENSITY AND FREQUENCY OF SARCOPENIA IN PRE-FRAIL NON-FALLERS, FALLERS AND RECURRENT FALLERS.

	**Pre-Frail (Total) (n=90)**	**Non-fallers (0 fall) (n=41)**	**Fallers (n=25)**	**Recurrent fallers (≥2 falls) (n=23)**	**p**
**T-Score ˣ**					
**T-Score L1-L4**	-1.19±1.57; -1.40 (-3.80; 3.00)	-1.21±1.57; -1.50 (-3.80; 2.70)	-1.16±1.39; -1.30 (-3.50; 1.50)	-1.20± 1.82; -1.5 (-3.80; 3.00)	0.91
**T-Score Femoral neck**	-1.37±0.94; -1.50 (-3.20; 1.50)	-1.31±0.93; -1.30 (-2.80; 1.50)	-1.38±1.06; -1.90 (-2.90; 1.30)	-1.47±0.87; -1.45 (-3.20; 0.80)	0.66
**T-score Fêmur**	-0.59±1.03; -0.80 (-2.80; 3.10)	-0.42±1.07; -0.50 (-2.50; 3.10)	-0.83±0.97; -1.15 (-2.00; 1.90)	-0.64±1.01; 0.75 (-2.80; 1.40)	0.13
**Densitometric classification Φ**					
**Normal BMD**	18.9 (17)	26.2(11)	16.0(4)	8.7(2)	0.18
**Osteopenia**	53.3 (48)	45.2(19)	68.0(17)	52.2(12)	
**Osteoporosis**	26.7 (24)	26.2(11)	16.0(4)	39.1(9)	
**Frequency of sarcopenia Φ**					
**No Sarcopenia**	78.9(71)	78.6(33)	72.0(18)	87.0(20)	0.67
**Probable Sarcopenia**	16.7(15)	19.0(8)	24.0(6)	4.3(1)	0.71
**Sarcopenia**	4.4(4)	2.4(1)	4.0(1)	8.7(2)	0.80
**Severe Sarcopenia**	-	-	-	-	-

Results described as ˣmean ±standard; median (minimum; maximum) compared by Kruskal Wallis test; and ΦRelative in percentage and absolute (number) frequencies compared by Chi-square or Fisher´s exact test; L1-L4, lumbar spine T-score mean (L1-L4 segment); BMD, bone mineral density.

APPENDIX 3: Figure 1. ROC curves.

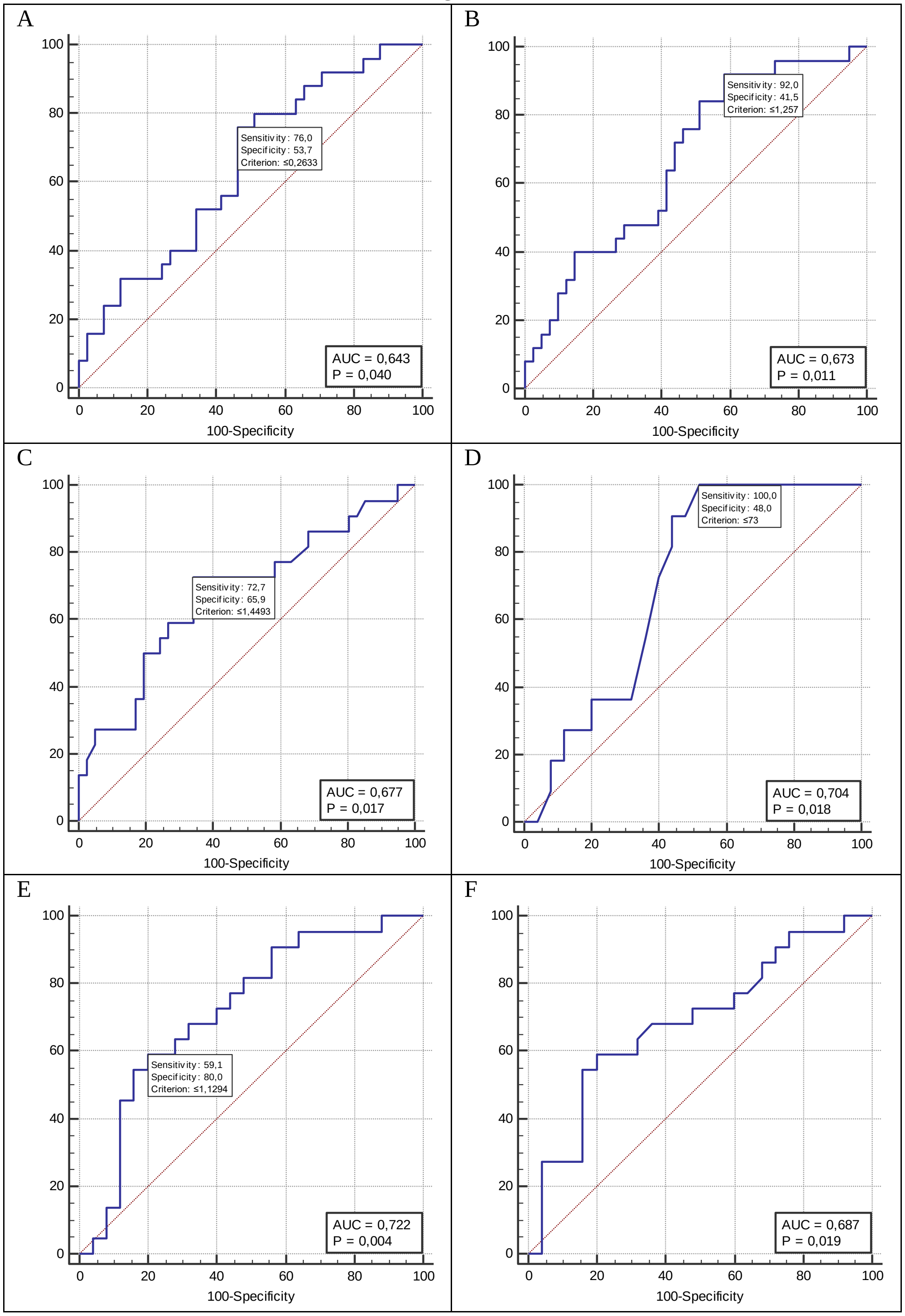

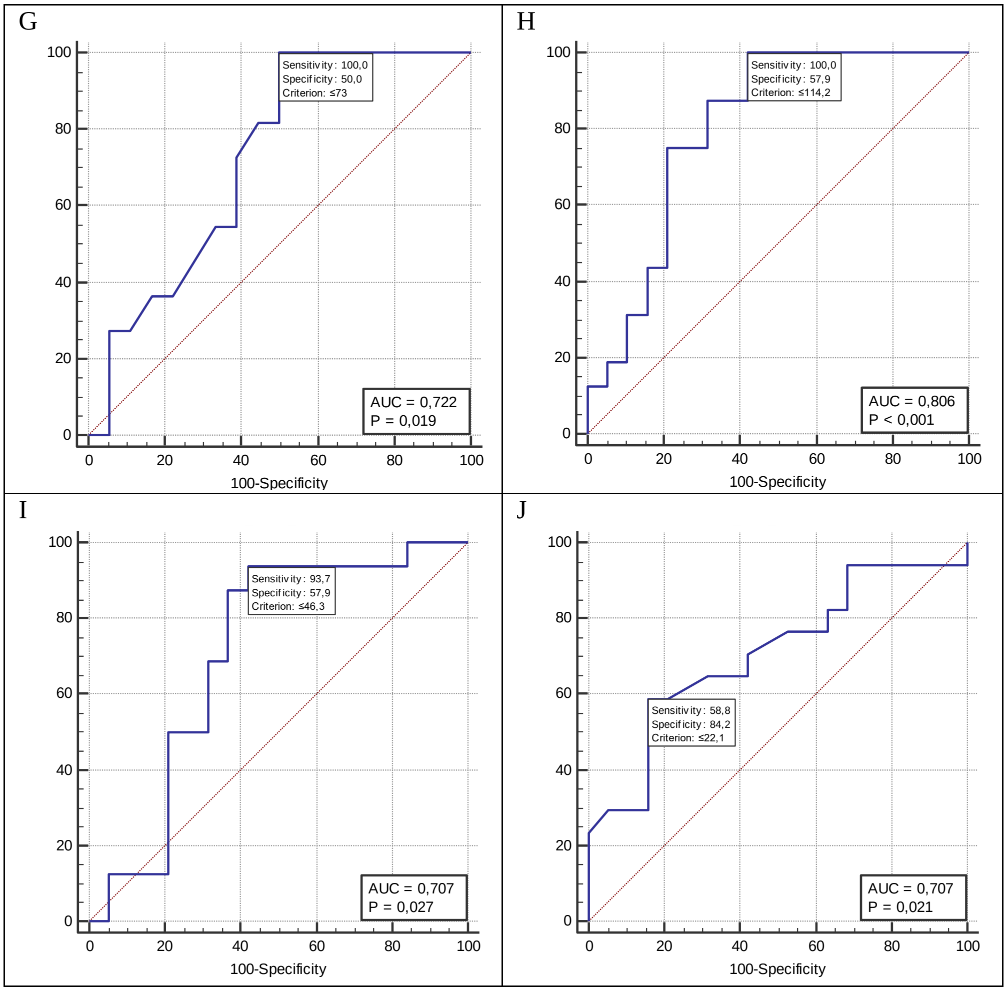

A (Walking speed reserve) and B (Walking speed ratio), corresponding to the significant outcomes for differentiating single fallers from non-fallers older women. C (Fast gait speed) and D (step length), corresponding to the significant outcomes to differentiate recurrent fallers from non-fallers older women. E (Usual gait speed), F (fast gait speed), G (Step length), H (Knee extension isometric peak torque), I (Knee flexion isometric peak torque) and J (Ankle dorsiflexion isometric peak torque), corresponding to the significant outcomes to differentiating recurrent fallers from fallers (single fall) older women.
